# Modification of Inclusions by Rare Earth Elements in a High-Strength Oil Casing Steel for Improved Sulfur Resistance

**DOI:** 10.3390/ma16020675

**Published:** 2023-01-10

**Authors:** Xueyuan Jiang, Gen Li, Haiyan Tang, Jinwen Liu, Sen Cai, Jiaquan Zhang

**Affiliations:** School of Metallurgical and Ecological Engineering, University of Science and Technology Beijing, No. 30 Xueyuan Road, Haidian District, Beijing 100083, China

**Keywords:** rare earth, oil casing steel, inclusions, thermodynamic calculation, sulfur resistance

## Abstract

Steel casing pipes used in the construction of deep oil wells usually require both high strength and corrosion-resistant behavior. Due to the exploration of deep H_2_S-bearing oil reservoirs, sulfide stress cracking (SSC) is becoming an increasingly serious concern for casing steel. The nonmetallic inclusions in the steel are among the key reasons for its service failure. The rare earth element Ce can be used to modify the inclusions in casing steel and improve its SSC resistance. Here, taking C110 grade casing steel (the highest class currently in service) as the investigated object, the modification behavior of Ce inclusions in the steel and the effect of the addition of Ce in varying amounts (0.01, 0.024, and 0.042 wt.%) on the modified products were studied through high-temperature tube furnace experiments and thermodynamic calculations. The results showed that Ce had an obvious modification effect on the CaO·Al_2_O_3_ inclusions in casing steel, and the diffusion of dissolved Ce in the steel was the limiting step of the modification reaction. With the extension of reaction time, the sequence describing the modification of inclusions in the steel was determined as follows: CaO·Al_2_O_3_ → CeAlO_3_ → Ce_2_O_3_/Ce_2_O_2_S. The final stable product after modification depended on the amount of Ce added. With 0.01 wt.% Ce, the stable phase in molten steel was Ce_2_O_3_; on the other hand, upon adding ≥0.024 wt.% Ce, the stable phase became Ce_2_O_2_S. In addition, the thermodynamic stability of Ce_2_O_3_ decreased, and it was transformed into CeAlO_3_, Ce_2_O_2_S, Ce_2_S_3_, and CeS during solidification. On the basis of our results and the considerations for smooth casting, the addition of a proper amount of a rare earth element is suggested for industrial trials, following the achievement of a significant and surprising improvement in the qualified rate of SSC resistance for the final steel products. The relevant mechanism is also analyzed.

## 1. Introduction

Casing pipes play a key role in reinforcing the walls of oil wells. There is currently an increasing demand for steel casing pipes with both higher strength and better corrosion-resistant behavior due to the need for even deeper oil and gas field exploration, which is accompanied by severe H_2_S media [1].

In a humid environment, H_2_S gas dissolves in water, reducing the environmental pH and forming an acidic atmosphere, which induces sulfide stress cracking (SSC) corrosion [2] and hydrogen-induced cracking (HIC) corrosion [3] in steel underground. Both SSC and HIC are, in essence, forms of hydrogen embrittlement caused by the interaction between hydrogen atoms and the heterogeneous matrix of steel [4]. Hydrogen atoms diffuse directly into the steel matrix and are captured by hydrogen traps, such as inclusions, grain boundaries, and dislocations in the steel. When the number of hydrogen atoms reaches a critical value, they combine to form hydrogen molecules, thus generating local hydrogen pressure. Subsequently, failure of the steel occurs below its yield strength due to the combined action of corrosion and tensile stress [5,6]. Relevant studies [7,8,9,10] have pointed out that the interface between inclusions and the steel matrix often becomes the source of cracks in the failure process, whereby the morphology and size distribution of inclusions directly affect the SSC resistance of the steel. Asadipoor et al. [7] reported that, due to the stress concentration caused by sharp and hard Al_2_O_3_ inclusions, cracks were produced at the interface between the Al_2_O_3_ inclusions and the matrix, which became hydrogen traps. In addition, small low-melting-point Ca–Al–O inclusions in the molten steel may aggregate into large inclusions during the continuous casting process. These large inclusions are deformed into a series of B-type inclusions during the subsequent rolling operation, which also become hydrogen traps and reduce the SSC resistance of the steel [8,9,10]. To avoid the influence of these harmful inclusions, researchers have aimed to remove them as best as possible in the process of steel production. However, due to the cost and productivity limitations of steelmaking, inclusions in steel cannot be completely removed. In this case, the characteristics of the inclusions are often controlled by modifying them so as to reduce their adverse effects on the steel. Commonly used methods include Ca treatment and slag modification.

Rare earth (RE) possesses the ability to purify molten steel, modify inclusions, and improve the solidification structure and properties of steel. In recent years, it has attracted extensive attention, becoming a research hotspot in the steel industry. The inclusions in steel modified by rare earth elements are similar to the steel matrix in terms of rigidity, hardness, toughness, brittleness, and thermal expansion. Furthermore, they more easily deform along with the steel matrix during rolling than other common inclusions [11]. Yang et al. [12,13,14,15] studied the inclusions in X80 pipeline steel modified by rare earth and indicated that an appropriate addition of rare earth elements was beneficial to improving the overall properties of the steel. On one hand, rare earth elements can modify harmful sharp Al_2_O_3_, long strip-like MnS, and other inclusions into tiny spherical rare earth oxysulfide inclusions, improving the mechanical properties of steel. On the other hand, rare earth elements can improve the compactness of corrosion products and protect the steel matrix from environmental erosion. According to their research, the optimum rare earth content in X80 steel was 0.0093 wt.% [15]. Zuo [16] found that, after adding 0.05 wt.% RE to X70 pipeline steel, MnO·SiO_2_ inclusions were modified to rare earth silicate composite inclusions, and the corrosion resistance of the steel was also enhanced. Yu et al. [17] added 0.011–0.023 wt.% Ce to ferritic stainless steel and revealed that the MnS and SiO_2_ inclusions were eventually transformed into Ce_2_O_3_·SiO_2_ and Ce_2_O_2_S fine inclusions. The addition of the rare earth element reduced the average interface area between inclusions and the steel matrix, thereby improving the pitting corrosion resistance of steel. Thus, SSC crack initiation was restrained.

The properties of final products in steel modified by rare earth are closely related to the addition quantity of rare earth element. Ma et al. [18] studied the inclusions in Ce-modified 55SiCr high-stress spring steel. When the addition of Ce was 0.02 wt.%, the inclusions in the steel mainly consisted of modified MnS, Ce–O–S, and Ce–S inclusions with sizes of 1–3 μm and a spherical or ellipsoid geometry. However, when the Ce addition was increased to 0.26 wt.%, the MnS inclusions disappeared, instead generating a large number of angular and irregular Ce–O and Ce–O–S inclusions over 5 μm in size, which affected the fatigue properties of the steel. To render these inclusions harmless, the appropriate quantity of rare earth element for addition to the steel was suggested to be 0.01–0.02 wt.%. Large-sized MgO·Al_2_O_3_ spinels presenting as D- or Ds-type inclusions often appear in steels, seriously deteriorating their fatigue life. Li [19] and Huang et al. [20] pointed out that these spinels could also be modified into rare earth inclusions by Ce to reduce their harmful nature according to the following modification route: MgO·Al_2_O_3_ → CeAlO_3_ + MgO → Ce_2_O_3_ + MgO → Ce_2_O_3_ [20]. In addition, the inclusions modified by rare earth can be used to refine the solidification structure. Ji et al. [21] added Fe–10 wt.% Ce alloy to Fe–4 wt.% Si molten alloy and found that the grain size of the initial δ-ferrite obviously decreased from 1906 μm to 1007 μm. In our previous study [22], it was found that the Ce_2_O_3_ particles generated upon the addition of Ce to TWIP steel could significantly refine the solidification structure. When 0.02–0.04 wt.% Ce was added, the ratio of equiaxial grain area for the as-cast solidification structure increased from 25% to 72%, the average equiaxial grain size decreased from 480 μm to 130 μm, and the microsegregation of alloy elements was improved.

In summary, rare-earth elements can effectively modify the inclusions in steel and possibly improve the SSC resistance of steel. However, there are considerable differences in the behavior mechanism and critical addition of rare earth elements for different steels. The C110 grade oil casing steel used in this study is a typical high-strength steel with a relatively high demand for sulfur resistance. It is mainly used to manufacture the casing pipes used in oil wells, which are thousands of meters of deep and contain sour H_2_S gas. At present, only two steel plants in China possess the production capacity for this type of steel. In the last two years, the statistics collected for Φ87 mm (6.5 mm thick) thin-walled steel pipe production in a steel plant have shown that the qualified rate of SSC resistance is less than 70%, which seriously influences production efficiency and the stability of product quality. In the present study, to further improve this quality index, the rare earth element Ce was added to C110 oil casing steel based on a delicate experimental analysis. The inclusion modification rule and mechanism in the C110 steel associated with Ce addition were studied using a tubular furnace experiment together with thermodynamic calculations. The proper quantity and time for Ce addition were determined for industrial applications. The qualified rate of SSC resistance of this steel in industrial tests increased to 91.7%, representing a significant and surprising effect.

## 2. Experimental Method

### 2.1. Tubular Furnace Study Method

C110 grade casing steel billet from a steel plant was used as the parent steel in our lab experiment; its chemical composition is shown in Table 1. The billet was produced via the industrial route using a 150 t electric-arc furnace (EAF), ladle furnace (LF), and vacuum degasser (VD) followed by Ca wire treatment and continuous casting (CC).

**Figure 1:**
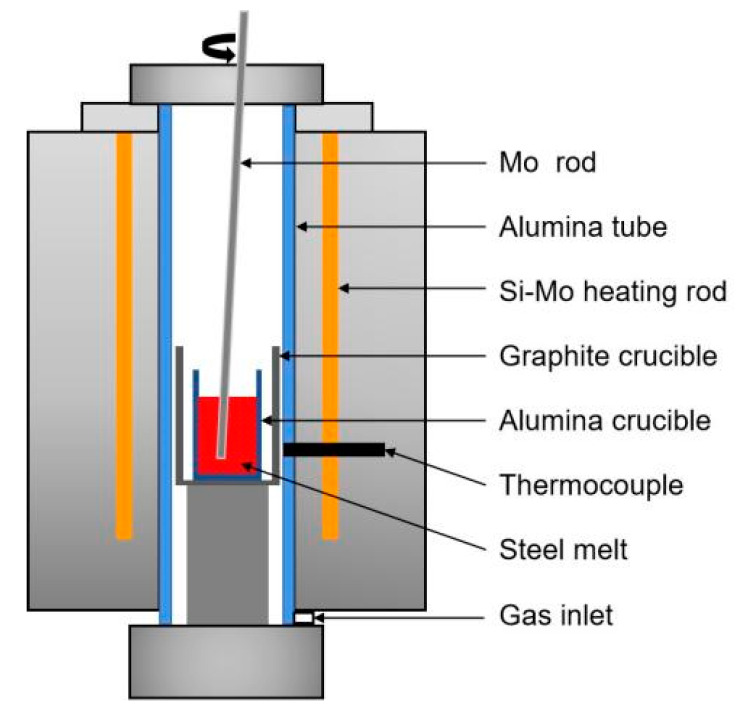
Structural diagram of the experimental tubular furnace.

**Figure 2:**
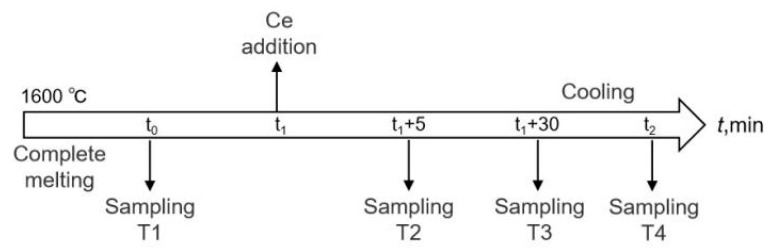
Schematics of rare earth addition and steel sampling times in experiment.

The rare earth additive adopted in this experiment was a self-made Fe–Ce–Si alloy, which was melted in a vacuum induction furnace (WZG-2 kg) with industrial pure Fe, pure Ce, and Si powder as raw materials. Its main chemical composition (mass fraction, %) included Fe (57.53), Si (16.57), and Ce (25.8). According to the amount of Ce added (0.01, 0.024, and 0.042 wt.%), the experiment was divided into three groups: L-Ce (low), M-Ce (middle), and H-Ce (high), respectively. The experimental device is shown in Figure 1. About 450 g of billet sample was cut in each furnace experiment. After removing the surface oil stains and iron oxide with a grinder, the sample was placed in the Al_2_O_3_ crucible in the constant temperature zone of the tubular furnace (SKL16- Φ80 × 250-12). The sample was heated to 1600 °C under argon protection at a heating rate of 1 °C/min. The experimental charging and sampling process is shown in Figure 2. After the molten steel completely melted, sample T1 was taken with a quartz tube 6 mm in diameter, then the designated amount of rare earth alloy was added to the molten steel. After stirring for 10 s with a molybdenum rod, samples T2 and T3 were taken individually from the molten steel at the set times, as shown in Figure 2. The samples were quenched and cooled for subsequent chemical composition and inclusion detection. The electric power was turned off immediately after sample T3 taken. When the molten steel was cooled to room temperature, the ingot (T4) was taken out to analyze the equilibrium transformation behavior of the inclusions during air cooling.

**Figure 3:**
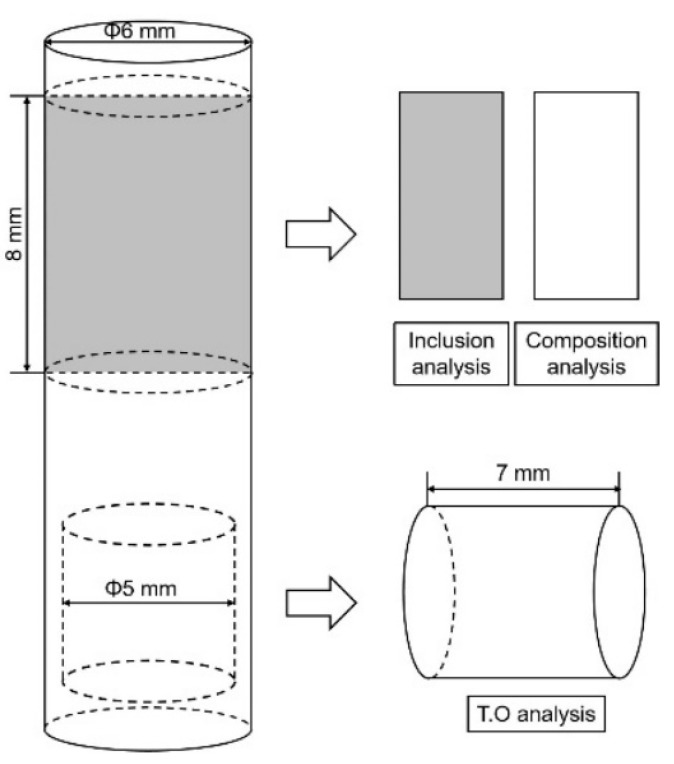
Schematic diagram of the processing of the molten steel sample.

The processing of the samples T1–T3 is shown in Figure 3. A small Φ5 mm × 7 mm rod was cut from each sample for the total oxygen (T.O) content analysis of the molten steel using an oxygen, nitrogen, and hydrogen analyzer (EMGA-830, Horiba, Kyoto, Japan). The remaining samples were cut along the centerline, where half was used for composition analysis and the other half for inclusion detection. An ICP-AES analyzer (OPTIMA 7000, PerkinElmer, Waltham, MA, USA) was used to test the contents of Als (acid soluble aluminum), Ca, and Ce in the steel, while the S content was measured with a carbon and sulfur analyzer (EMIA-820, Horiba). The characteristics of the inclusions were observed using a scanning electron microscope (FE-SEM) equipped with an energy dispersive spectrometer (EDS, Thermo Ns7, Waltham, MA, USA). The ingots (T4) were cut 30 mm away from the bottom, and samples were taken in a similar way for chemical composition detection and inclusion analysis. The amount of rare earth alloy added and the measured composition of molten steel for each sample are shown in Table 2, in which the contents of Als, Ca, T.O, and S are the average values of the T1–T4 samples.

### 2.2. Industrial Trial Study Method

Based on the abovementioned laboratory study, an industrial trial was performed. The production route was still 150 t EAF–LF–VD–Ca treatment–CC. Twenty-one kilograms of RE–Fe alloy (main chemical composition: Ce ≥ 38 wt.%, La ≥ 19 wt.%, Fe remaining) was added after Ca treatment, followed by soft argon stirring for 15 min to allow for casting. The amount of RE added was about 0.0080 wt.% in the molten steel. The main chemical composition of the steel billet was measured; the results were basically the same as those in Table 1, where the total RE content was 0.0055 wt.% (0.0035 wt.% Ce + 0.0020 wt.% La). The billets with and without RE were sampled for inclusion observation. The sulfide stress corrosion resistance of corresponding pipe samples was compared between those with and without rare earth alloy, according to method A of NACE TM 0177-2005. The pipe sample was processed into a Φ5 mm × 100 mm bar, which was then soaked in saturated H_2_S solution for 720 h under a specified load (85% minimum yield strength). The steel was considered to be of good quality if no fracturing occurred during this process.

## 3. Experimental Results

### 3.1. Tubular Furnace Experiment Results

#### 3.1.1. Morphology and Composition of Inclusions in Steel before Rare Earth Addition

**Figure 4:**
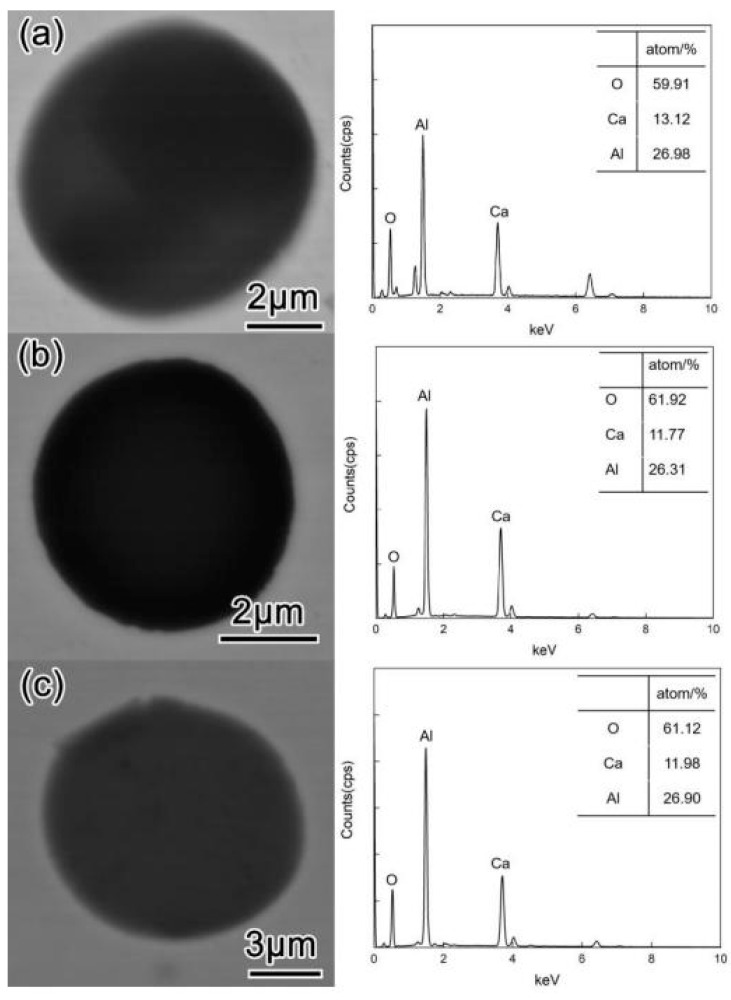
Typical morphology of inclusions in steel before adding rare earth alloy (T1): (**a**) L–Ce; (**b**) M–Ce; (**c**) H–Ce.

The casing steel billet used in this experiment was deoxidized with Al and treated with Ca during industrial operation. Therefore, before adding the Ce alloy (at T1), the main inclusions in the three groups of samples were spherical calcium aluminate, as shown in Figure 4. The EDS results showed that the atomic ratio of Ca to Al in the inclusions was about 1:2–2.5. Thus, they were speculated to be CaO·Al_2_O_3_ inclusions. The melting point of this kind of inclusion was 1605 °C, meaning they were in a liquid state at the steelmaking temperature but were not easily deformed during rolling [8]. Therefore, these inclusions were likely to become the source of cracks due to stress concentration [23]. As shown in Table 2, during heating and melting, the contents of Als and Ca in the samples were significantly lower than those in the raw billet materials; however, the T.O content increased from 0.0018 wt.% to over 0.0024 wt.%. The reason for the former may be that the Al and Ca in the molten steel were oxidized during the experimental heating, while the increase in T.O content was the result of air inhalation.

#### 3.1.2. Morphology and Composition of Inclusions in Steel during Rare Earth Modification

**Figure 5:**
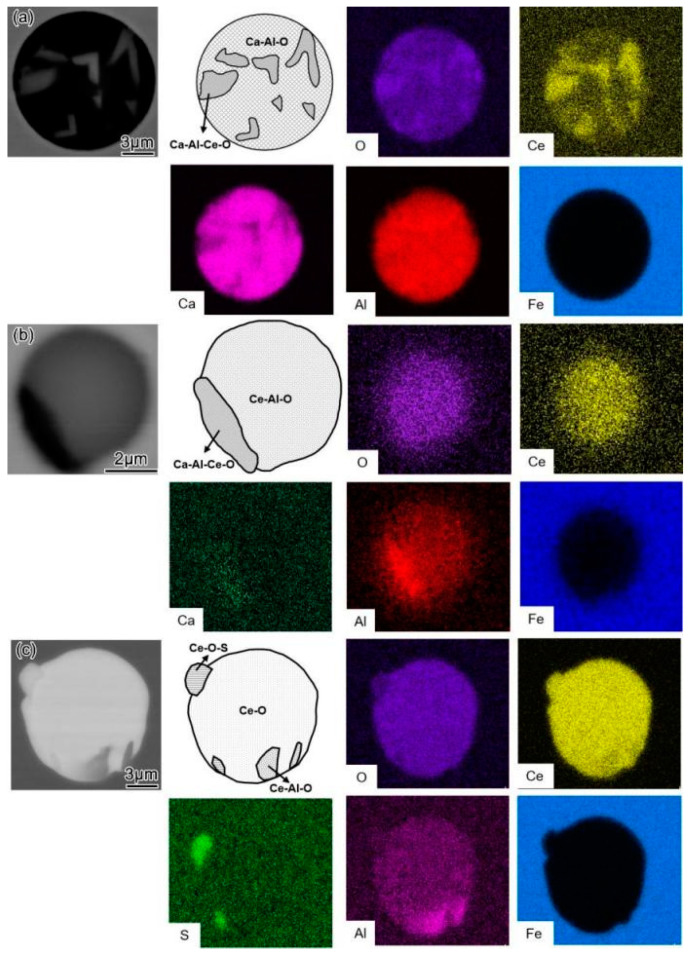
Morphology of typical inclusions in steel after reaction with Ce for 5 min (T2): (**a**) L–Ce; (**b**) M–Ce; (**c**) H–Ce.

After adding the Ce alloy to the molten steel and allowing the reaction to proceed for 5 min (T2), the CaO·Al_2_O_3_ inclusions were transformed into inclusions containing Ce. However, the degree of modification varied according to the Ce content. For the L–Ce sample, where the amount of Ce added was 0.01 wt.%, the typical morphology of the steel inclusions is shown in Figure 5a. The main body of the inclusion was still a black spherical Ca–Al–O system, while in some areas of the inclusion, the Al was replaced by Ce, forming a white Ca–Al–Ce–O composite inclusion. In the M–Ce sample, where the addition of Ce was increased to 0.024 wt.%, the inclusion was mostly transformed into a Ce–Al–O system with a small amount of Ca–Al–Ce–O on the outer side, as shown in Figure 5b. In the H–Ce sample, the addition of Ce was further increased to 0.042 wt.%. After 5 min of reaction time, the inclusions exhibited mixed phases of Ce–Al–O, Ce–O, and Ce–O–S, as shown in Figure 5c. In addition, Ca was not detected, indicating that Ca was completely reduced by Ce.

**Figure 6:**
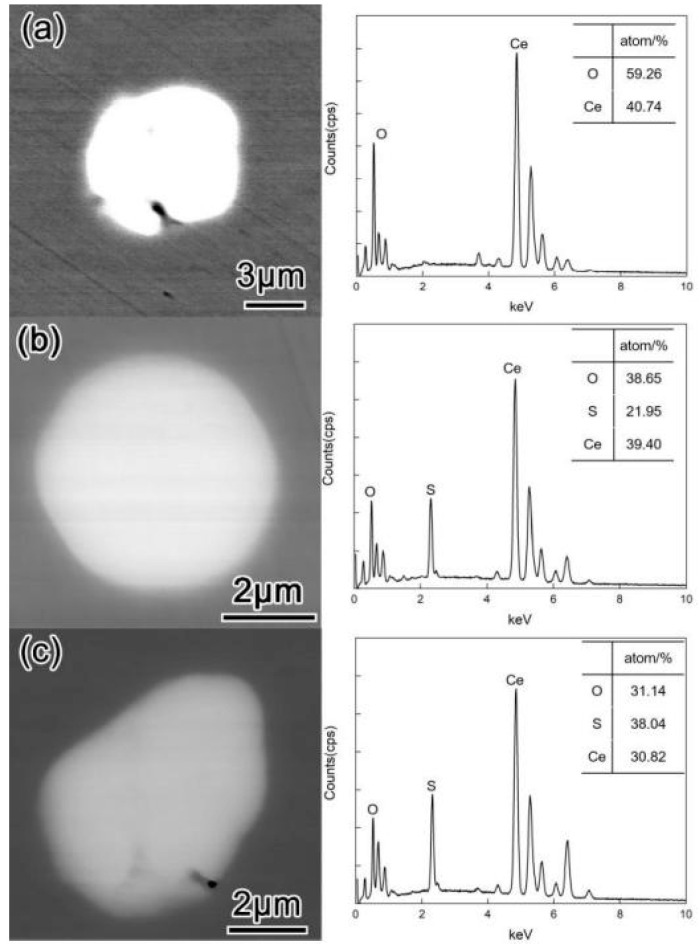
Morphology of typical inclusions in steel after reaction with Ce for 30 min (T3): (**a**) L–Ce; (**b**) M–Ce; (**c**) H–Ce.

Figure 6 shows the morphology and composition of typical inclusions in the steel after adding Ce alloy for 30 min (T3). At this time, the CaO·Al_2_O_3_ inclusions were completely modified into Ce–O or Ce–O–S systems, and the Ca–Al–Ce–O and Al–Ce–O observed at T2 disappear. The inclusions in the L–Ce samples were Ce–O systems, while those in the M–Ce and H–Ce samples were Ce–O–S systems. Therefore, it can be considered that under the present experimental conditions and the amount of Ce added, the CaO·Al_2_O_3_ liquid inclusions are finally modified into Ce–O or Ce–O–S inclusions, while inclusions such as Ca–Al–Ce–O and Ce–Al–O are only found as the intermediate products in the modification process. This result also implies that the rare earth alloy should stay in the molten steel for a shorter time if Ca–Al–Ce–O and Ce–Al–O inclusions are desired.

**Figure 7:**
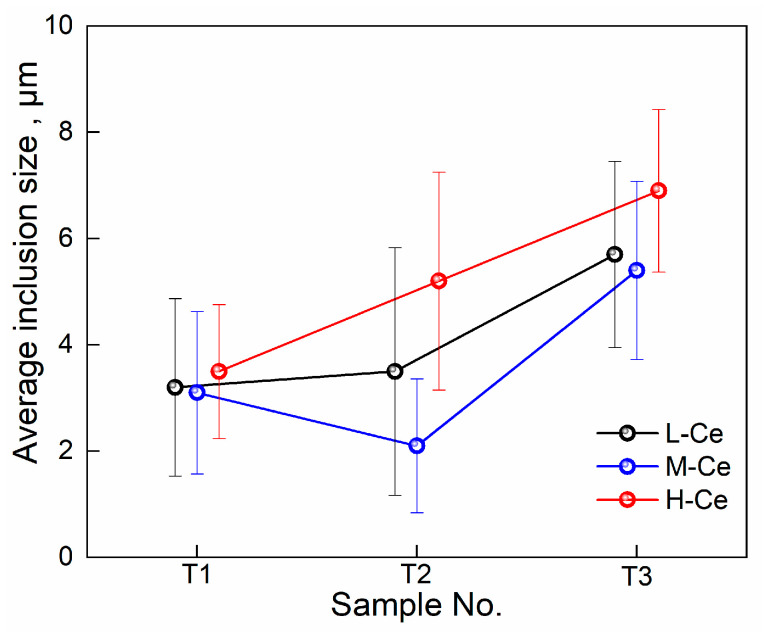
Statistical results for inclusion size.

To compare the effects of Ce content and heat holding time on inclusion size, inclusions greater than 1 μm in size and over 20 in numbers were counted using SEM for each sample. The results are shown in Figure 7, where the error bars were calculated using standard deviation. Before adding Ce (T1), the CaO·Al_2_O_3_ inclusions detected in each sample were 2~8 μm in size, with an average of 3.2 μm. At T2 (5 min after Ce addition), the inclusions in different samples varied greatly. The average size of inclusions in the M–Ce case decreased to 2.1 μm, while that in the H-Ce case increased to 5.2 μm. With the extension of holding time, the inclusion size at T3 (30 min after Ce addition) increased in all cases compared with that at T2, reaching 5.7, 5.4, and 6.9 μm for the different cases. These results indicate that the inclusions modified by Ce gradually grow and evolve with holding time, and their size is affected by the amount of Ce added.

#### 3.1.3. Morphology and Composition of Inclusions in Steel after Air Cooling

**Figure 8:**
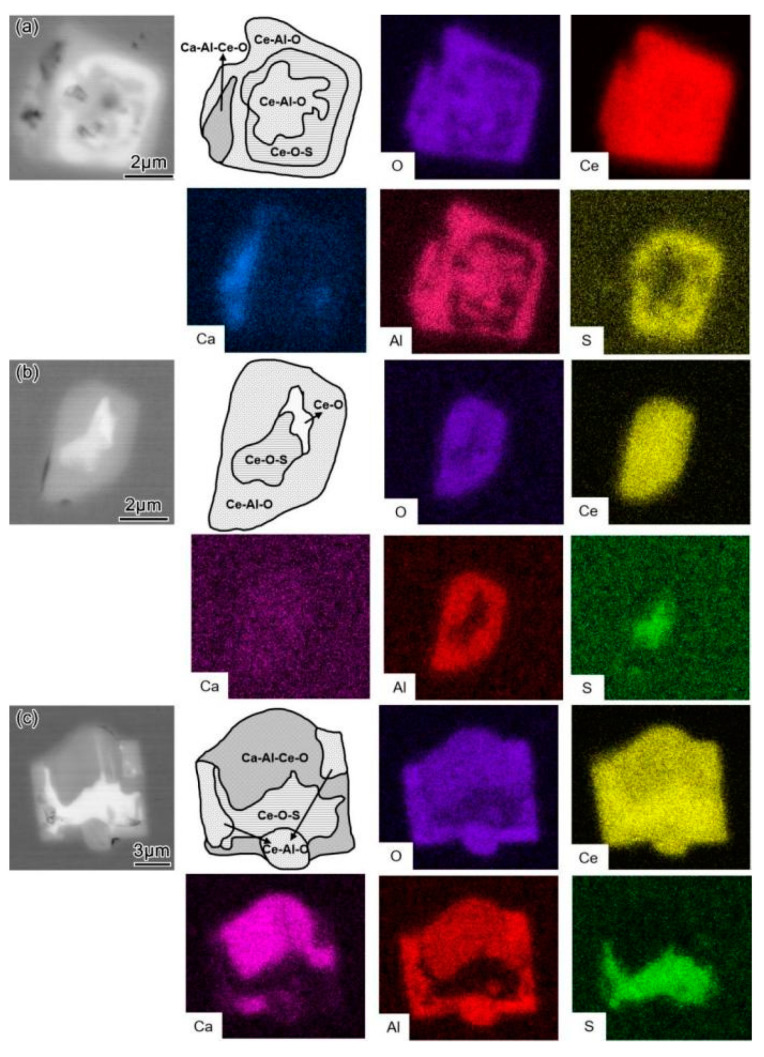
Typical inclusion morphology in ingots after steel solidification (T4): (**a**) L–Ce; (**b**) M–Ce; (**c**) H–Ce.

Typical inclusions in the ingots after cooling with the furnace (T4) are shown in Figure 8. Compared with T3, the composition of the inclusions changed again at T4 with cooling from simple Ce–O or Ce–O–S inclusions to complicated composite inclusions. In the L–Ce sample, the inclusions changed from Ce–O at T3 to Ce–Al–O + Ca–Al–Ce–O + Ce–O–S at T4, as shown in Figure 8a. In the M–Ce sample, the inclusions were composed of a core of Ce–O–S + Ce–O and an outer layer of Ce–Al–O (Figure 8b), where the Ce–Al–O layer was formed during cooling. The inclusions in the H–Ce sample were mainly composed of Ce–O–S in the core, Ca–Al–Ce–O in the outer layer, and a small amount of Ce–Al–O, as shown in Figure 8c.

### 3.2. Industrial Trial—Results of Inclusion Observation and Sulfur Resistance Test

**Figure 9:**
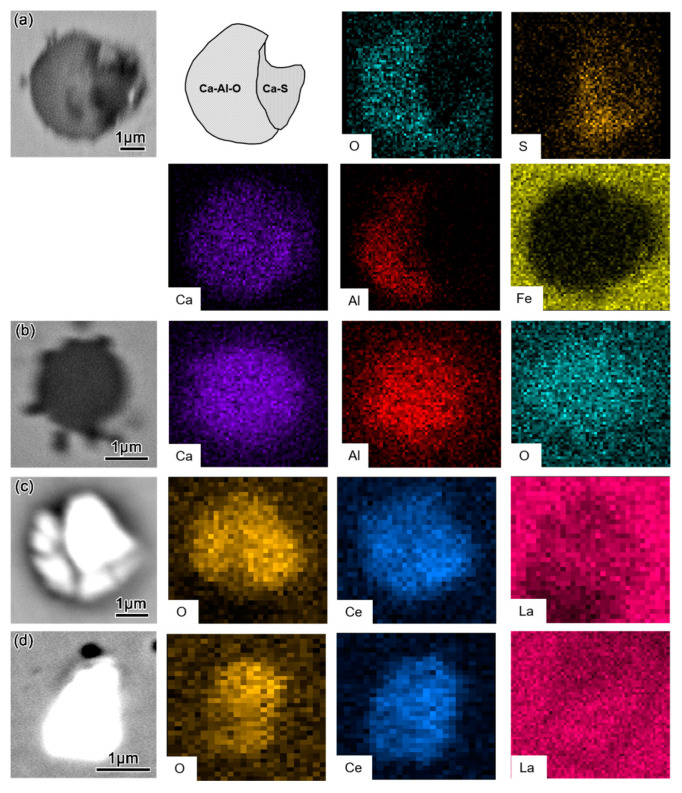
Typical morphology of inclusions in industrial billet (**a**,**b**) without a rare earth compound and (**c**,**d**) with the addition of a rare earth compound.

Figure 9a,b show the typical morphology of inclusions in the cast billet of C110 grade casing steel without rare earth alloy in the industrial trial. The inclusions in the molten steel were mainly Ca–Al–O together with a small amount of CaS and were typically spherical or nearly spherical in shape. However, after rare earth treatment, the inclusions in the steel were mainly composed of Ce–La–O compounds, as shown in Figure 9c,d. It can be observed that the Ca–Al–O and CaS + Ca–Al–O inclusions were modified into Ce–La–O inclusions in industrial production due to the appropriate addition of the rare earth compound. These results are basically in agreement with those of the L–Ce case in the tubular furnace experiment. It should be noted that we added a commercially made Ce–La–Fe alloy in the industrial trial, while a self-made Ce–Fe alloy was adopted for the tubular furnace experiment in our laboratory (the Ce–Fe alloy could not be obtained from the market at the time of our industrial trial). As the chemical properties and atomic weight of La are very close to those of Ce, the modification effect of the two elements on steel should be similar, with negligible differences.

**Figure 10:**
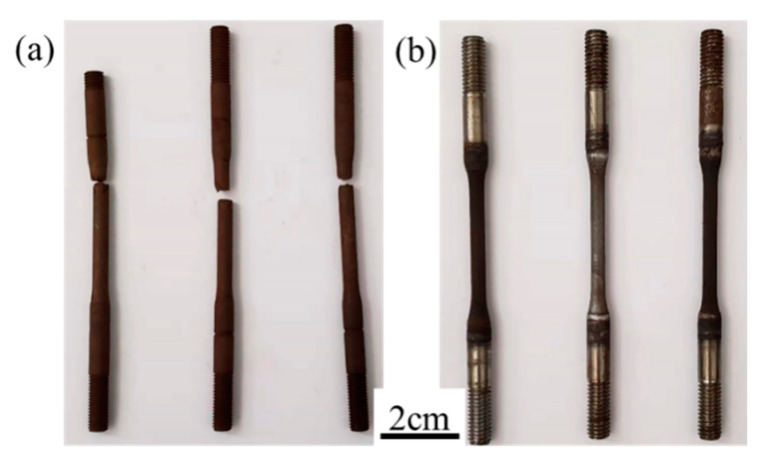
Casing steel sample morphology after SSC resistance test (NACE A method) (**a**) without RE (**b**) and with RE addition (0.008 wt.%).

The typical morphology after the SSC resistance test is shown in Figure 10. The results showed that almost half of the samples that did not undergo rare earth treatment broke at less than 720 h, while those treated with rare earth were all intact, i.e., the qualified rate of SSC corrosion resistance was increased to 100% for this test. According to the statistics from the three industrial trials with rare earth addition, the comprehensive qualification rate of Φ87 mm (6.5 mm thick) in C110 thin-walled pipe for sulfide stress corrosion resistance increased from an average of 66% to 91.7%, representing a significant and surprising positive effect.

## 4. Analysis and Discussion

### 4.1. Analysis of Inclusion Modification Process in Tubular Furnace

According to the above experimental results and a review of the literature [24,25], Ce will combine with O, S, and other elements after being added into molten steel; in addition, it will also modify the original Ca–Al–O system inclusions into CeAlO_3_, Ce_2_O_3_, and Ce_2_O_2_S. The standard Gibbs free energy of the relevant reactions is shown in Table 3.

To compare the stability of the reaction products after the addition of Ce to molten steel at T1 under our experimental conditions, the activity of [Ce], [Al], [O], [S], and other elements in the steel was calculated using Formula (7):

(7) $$\log a_{i}=\log[\%i]+\sum (e_{i}^{j}\left[\%j\right]+r_{i}^{j}\left[\%j{]}^{2}+r_{i}^{j,k}[\%j{]}^{2}[\%k{]}^{2}\right)$$

where $a_{i}\;$ is the activity of element *i*; $e_{i}^{j}$ is the interaction coefficient of element *j* on *i*; $r_{i}^{j}$ is the second-order interaction coefficient of element *j* on *i* in molten steel; $r_{i}^{j,k}\;$ is the second-order cross interaction coefficient of element *j* and *k* on *i*; and $\left[\%i\right]$, $\left[\%j\right]$, and $\left[\%k\right]$ are the mass percentage concentrations of the elements *i*, *j*, and *k* in molten steel, respectively. In the calculation process, we adopted a concentration of 1% as the standard state. The relevant parameters are shown in Table 4.

In addition, the O content (shown in Table 2) represents the total oxygen in the steel; however, the calculation of Formula (7) requires the dissolved oxygen ([O]) content. Therefore, the [O] before the addition of Ce was calculated according to the Al–O reaction equilibrium in steel (Equation (8)) [32]:

(8) $$\left[\mathrm{Al}\right]+\left[O\right]={\left({\mathrm{Al}}_{2}O_{3}\right)}_{\mathrm{inclusion}}{\;\Delta G}^{\theta }=-1,225,417+393.86\;T$$

where the activity of the Al_2_O_3_ inclusion was selected as 0.2467 according to reference [33]. The calculation results showed that the [O] content was about 0.0008 wt.%.

**Figure 11:**
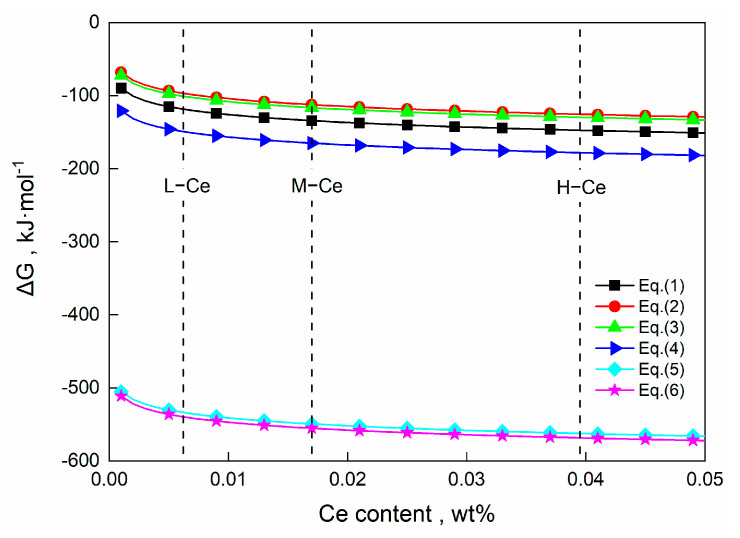
Relationship between Gibbs free energy of Ce modification reaction in molten steel and Ce content at 1600 °C.

Figure 11 shows the effect of Ce addition on the Gibbs free energy of the reactions in Formulas (1)–(6) at 1600 °C. It can be seen that the Gibbs free energy of each reaction had a negative value, indicating that from the perspective of thermodynamics, all six reactions could occur after Ce addition. Meanwhile, these results also showed that Ce could easily modify CaO·Al_2_O_3_ into CeAlO_3_, Ce_2_O_3_, and Ce_2_O_2_S (as shown in Formulas (4)–(6)). Therefore, inclusions containing the three phases were observed in the samples taken at T2.

**Figure 12:**
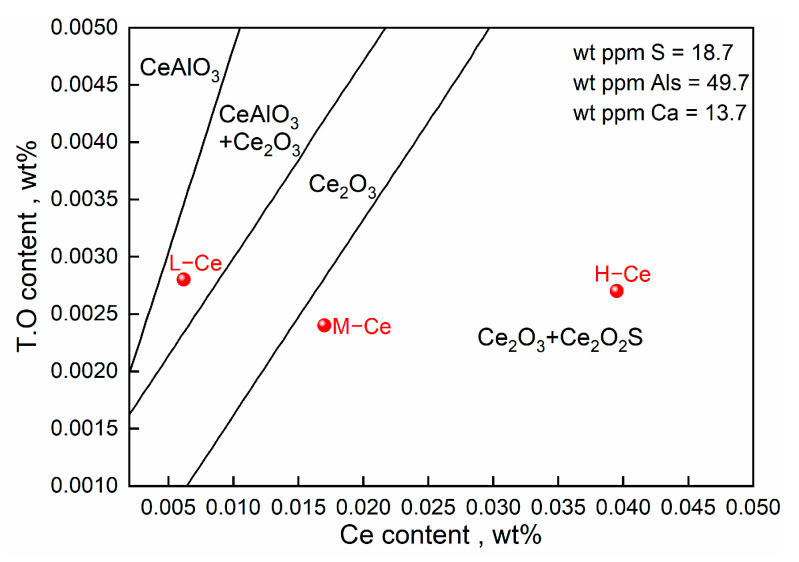
Stable phase diagram of Ce–Al–O–S inclusions in molten steel at 1600 °C.

To further analyze the evolution mechanism of inclusions in steel after Ce addition, a stable phase diagram of the Ce–Al–O–S system at T3 and 1600 °C was generated using the phase diagram module in the FactSage 8.1 thermodynamic software. The selected databases included FactPS, FToxide, FTmisc, and SGPS. The main chemical composition of the molten steel was taken from Table 1 and the contents of Ca, Als, and S were obtained from the average of the three schemes. The calculation results are shown in Figure 12, in which the red dots represent the T.O and Ce contents of molten steel in the three experimental cases.

It can be seen that with an increase in Ce content in steel, Ce modified products in an equilibrium state are first converted from CeAlO_3_ to Ce_2_O_3_, and then to Ce_2_O_2_S. The reaction equation can be expressed as Formulas (9) and (10):

(9) $$\left[\mathrm{Ce}\right]{+\mathrm{CeAlO}}_{3}{=\mathrm{Ce}}_{2}O_{3}+\left[\mathrm{Al}\right]$$

(10) $${\mathrm{Ce}}_{2}O_{3}+\left[S\right]={\mathrm{Ce}}_{2}O_{2}S+[O]$$

It should be noted that the thermodynamic calculation in Figure 10 shows that the reaction products of the experimental samples were in the CeAlO_3_ + Ce_2_O_3_ and Ce_2_O_3_ + Ce_2_O_2_S two-phase regions in the equilibrium state, while only Ce_2_O_3_ and Ce_2_O_2_S inclusions were detected in the samples at T3. On one hand, this may be related to the limited number of inclusions detected. On the other hand, it may be because Ce_2_O_2_S formed in the late stage of the reaction wraps the surface of Ce_2_O_3_, resulting in the failure of the inclusion core to be detected.

**Figure 13:**
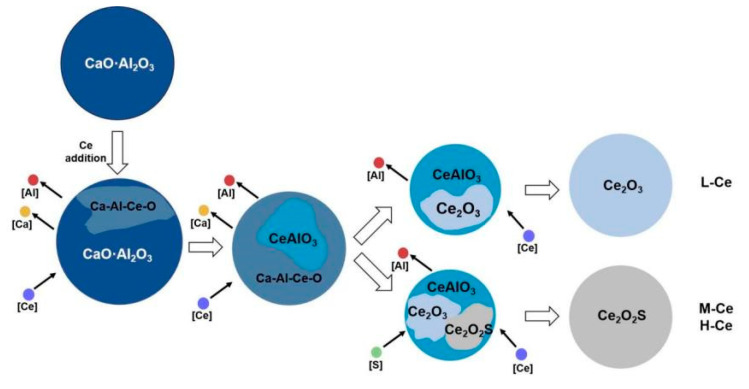
Schematics of modification of Ca–Al–O system inclusions by Ce in C110 steel.

As shown in Figure 5, the degree of inclusion modification differed with the different Ce contents at T2 (Ce alloy added for 5 min). In other words, the rate of the inclusion modification reaction varied between the three schemes. According to a study by Yu [17] and Li [34] et al. on the effect of different Ce concentrations on the modification of SiO_2_ and Al_2_O_3_ inclusions, the concentration difference in dissolved Ce ([Ce]) between the original inclusion interface and the unreacted core interface was the main reason for different modification rates. In this study, all other conditions were almost the same except for the Ce content. It can be seen from Figure 12 that the inclusion composition changed significantly with the increase in Ce content. This shows that during the modification of Ca–Al–O inclusions, the diffusion dynamics of [Ce] also represent a limiting step that affects the type of modified products. The process of the modification of Ca–Al–O system inclusions by Ce is clearly illustrated in Figure 13.

In the initial stage of smelting (t < 5 min), the Ca–Al–O system inclusions in the steel are reduced by Ce to generate Ca–Al–Ce–O system composite inclusions and CeAlO_3_. Meanwhile, the [Ce] content around the inclusions is reduced. Under the current experimental conditions, Ca–Al–Ce–O and CeAlO_3_ inclusions could not exist stably in the molten steel. With the extension of reaction time and the diffusion of [Ce], the reactions shown in Formulas (5), (6), (9), and (10) were allowed to occur; thus, the inclusions were further modified into Ce_2_O_3_ (L–Ce) and Ce_2_O_2_S (M–Ce and H–Ce). This is basically consistent with the results of the experimental trial. Therefore, in the industrial production process, argon blowing treatment should be adopted together with other measures to accelerate the dissolution of the alloy and improve the distribution uniformity of the rare earth element in the molten steel after it is added in the ladle during refining, which will promote the prompt completion of the modification reaction.

In addition, Figure 7 shows that in the M–Ce case, the average size of the inclusions decreased from 3.1 μm in T1 to 2.1 μm in T2 during the modification from liquid Ca–Al–O to Ca–Al–Ce–O and CeAlO_3_. This shows that adding an appropriate amount of Ce to molten steel is conducive to the formation of fine and dispersed inclusions; the same phenomenon was also reported in reference [35]. With the further conversion of CeAlO_3_ to Ce_2_O_3_ and Ce_2_O_2_S, the inclusion size increased, which was related to the increase in inclusion growth rate caused by the increase in [Ce] concentration, as shown in Formula (11) [36]:

(11) $$\frac{R^{2}}{t}=D\left(\frac{c_{0}-c_{e}}{2c_{p}}\right)$$

where *R* is the radius of the inclusions, *t* is the time, D is the solute diffusion coefficient, $c_{0}$ is the initial concentration of reactive elements in the molten steel, $c_{e}$ is the equilibrium concentration of reactive elements in the molten steel, and $c_{p}$ is the concentration of elements in the inclusions. An increase in Ce addition will increase $c_{0}$, while the extension of holding time will reduce $c_{e}$; both together will cause increases in inclusion growth rate and size.

### 4.2. Analysis of Inclusions during Cooling

**Figure 14:**
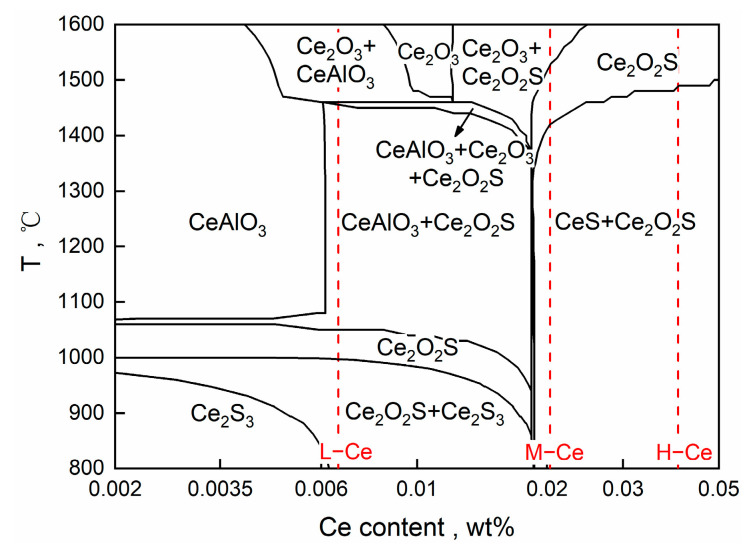
Simulation results of equilibrium precipitation of inclusions during ingot cooling.

Ren et al. [37,38] pointed out that the composition of inclusions will also change during the solidification and cooling of steel and the heat treatment process. The inclusions formed during this period will remain in the solid steel, affecting its service performance. Therefore, the transformation behavior of inclusions during cooling was simulated using the phase diagram module of the FactSage software 8.1. The basic chemical composition of the steel was taken from Table 1, and the contents of Als, Ca, O, and S were obtained from the average of the three schemes. The selected databases included FactPS, FToxide, FSstel, and SGPS. Compared with the FTmisc database, the FSstel database can better simulate the thermodynamic behavior of inclusions during solidification. The simulation results are shown in Figure 14.

It can be seen that the stability of the Ce-modified product, Ce_2_O_3_, decreased with the decrease in temperature, and some was transformed into CeAlO_3_, Ce_2_O_2_S, Ce_2_S_3_, CeS, and other was transformed into rare earth sulfides and oxy-sulfides. The reason for this is related to the decrease in the solubility of Al and S in molten steel with solidification. The corresponding reactions can be expressed as Formulas (12)–(15). During the temperature drop, in addition to the reaction between Ce and the precipitated [S], transformation reactions may also occur, as expressed by Formulas (16) and (17) [27]. Therefore, the inclusions observed at T4 in the L–Ce and M–Ce cases contained multiple phases of Ca–Al–Ce–O, Ce–Al–O, Ce–O, and Ce–O–S, as shown in Figure 8a,b. The thermodynamic calculations showed that there was no Al in the reaction products during the cooling process associated with the equilibrium state for the H–Ce case. However, Ca–Al–Ce–O inclusions were observed (Figure 8c), which may be due to the actual reaction not reaching equilibrium. This also reflects the limitations of thermodynamic software calculations.

(12) $${\mathrm{Ce}}_{2}O_{3}+\left[\mathrm{Al}\right]=\left[\mathrm{Ce}\right]+{\mathrm{CeAlO}}_{3}$$

(13) $${2\mathrm{CeAlO}}_{3}+[S]={\mathrm{Ce}}_{2}O_{2}S+{\mathrm{Al}}_{2}O_{3}$$

(14) $${\mathrm{Ce}}_{2}O_{2}S+2\left[S\right]={\mathrm{Ce}}_{2}S_{3}+2\left[O\right]$$

(15) $${\mathrm{Ce}}_{2}O_{2}S+\left[S\right]=2\mathrm{CeS}+2\left[O\right]$$

(16) $${2\mathrm{CeAlO}}_{3}+\mathrm{CaS}={\mathrm{Ce}}_{2}O_{2}S+{\mathrm{CaO}+\mathrm{Al}}_{2}O_{3}$$

(17) $${\mathrm{Ce}}_{2}O_{2}S+2\mathrm{CaS}={\mathrm{Ce}}_{2}S_{3}+2\mathrm{CaO}$$

In conclusion, the composition of inclusions containing Ce will change during solidification, combining with S in molten steel to reduce its activity and further helping to avoid the formation of CaS + Ca–Al–O duplex inclusions. CaS-bearing inclusions can degrade the castability of steel on account of its high melting point and lead to the erosion of the ladle slide gate and refractory nozzles during casting [39]. In addition, the CaS-rich layer is easily separated even from the core of the inclusions during the rolling process, which will likely cause a crack [40]. Therefore, adding Ce to steel can inhibit the formation of CaS during solidification, which should improve the SSC resistance and castability of the steel.

### 4.3. Design Basis of Rare Earth Addition and Analysis of Industrial Trial

Li et al. [41] observed the deformation of the steel matrix around inclusions after steel was stressed and found that rare earth inclusions were more consistent with the deformation of the steel matrix than Al_2_O_3_ inclusions. According to their theoretical calculations, the physical properties of rare earth inclusions, including Young’s modulus and Poisson’s ratio, were closer to those of the steel matrix than Al_2_O_3_ and CaO·Al_2_O_3_ inclusions in molten steel. In addition, rare earth inclusions can be used to refine the solidification structure as good nucleation cores for ferrite [21,22]. The lattice mismatch of Ce-modified products in steel based on Formula (18) [42] is shown in Table 5.

(18) $$\delta_{{\left(hkl\right)}_{n}}^{{\left(hkl\right)}_{s}}=\frac{1}{3}\overset{3}{\underset{i=1}{\sum }}\frac{\left|d_{\left[uvw\right]}{}_{s}^{i}\cos\theta -d_{\left[uvw\right]}{}_{n}^{i}\right|}{d_{\left[uvw\right]}{}_{n}^{i}}$$

In Formula (18), δ is the lattice mismatch; $d_{{\left[uvw\right]}_{s}^{i}}$ and $d_{{\left[uvw\right]}_{n}^{i}}$ are the atomic spacing between the nucleating substrate and the solidified phase in the direction of the [uvw] crystal, respectively; and *θ* is the angle between the two vectors.

It is generally believed [42,43] that the nucleating substrate has a good heterogeneous nucleation effect when the δ-value is less than 6%, while it may or may not have a heterogeneous nucleation effect when the δ-value is between 6 and 12%. As shown in Table 5, the δ-values of the Ce-modified products in the steel—Ce_2_O_3_, Ce_2_O_2_S, and CeS—were all less than 6%; thus, they met the requirements for being heterogeneous nucleation cores. CeAlO_3_ theoretically can act as a nucleation core, with a δ-value between 6 and 12%. However, there are no relevant reports on using it to refine the primary ferrite phase. Meanwhile, according to the research of Liu et al. [45], Ce_2_O_2_S is slightly brittle compared to Ce_2_O_3_. Therefore, Ce_2_O_3_ is more suitable as the target product of inclusion modification. Our experimental results showed that when Ce was added at 0.01 wt.% (L–Ce), the Ca–Al–O inclusions could be modified to form Ce_2_O_3_ under rapid cooling. In addition, considering the difference between the compositions of experimental (Table 2) and industrial (Table 1) steel, as well as the loss of Ce content in molten steel due to the floating of its inclusions or secondary oxidation, we added about 0.008 wt.% rare earth element to the C110 grade oil casing steel for the industrial test, with the intention of modifying the inclusions in the steel.

The results of the industrial test based on the above analysis were described in Section 3.2. These findings, in turn, prove the validity of the experimental design and the rationality of the mechanism analysis. On one hand, the generated rare earth inclusions possess physical properties that are more similar to those of the steel matrix than Al_2_O_3_ and CaO·Al_2_O_3_ inclusions, which will alleviate the stress concentration [41]. On the other hand, these inclusions can combine with S and Al in molten steel during solidification, which reduces the generation of CaS inclusions. Therefore, the SSC resistance performance of casing steel will be enhanced accordingly. In addition, these rare earth inclusions will improve the mechanical properties of steel due to the refinement of the solidification structure. The relevant industrial trials are still ongoing, and their findings will be reported in the future.

In summary, the addition of an appropriate amount of a rare earth element into high-strength oil casing steel will greatly enhance its sulfide corrosion resistance, especially when it is utilized under ultradeep and H_2_S-bearing oil wells. This study provides a promising application prospect for rare earth elements in quality steel products for corrosive oil and gas field exploration.

## 5. Conclusions

The modification of the inclusions in a high-strength and corrosion-resistant C110 oil casing steel by the rare earth element Ce was studied experimentally, and the modification mechanism was analyzed using thermodynamic simulation and production tests. Accordingly, an inclusion modification route using rare earth elements in the oil casing steel production process was realized with a considerable improvement in the sulfur resistance index of its products, and the following conclusions are drawn:

(1) With the extension of reaction time in molten steel upon the addition of Ce, the sequence for the modification of Ca–Al–O inclusions in C110 oil casing steel by Ce is CaO·Al_2_O_3_→CeAlO_3_→Ce_2_O_3_/Ce_2_O_2_S, and the stability of the final product depends on Ce content. Under the current experimental conditions, when the Ce content was 0.0062 wt.% (L–Ce, Ce addition 0.01 wt.%), Ce_2_O_3_ was the stable phase in molten steel; when Ce content reached 0.017 wt.% (M–Ce, Ce addition 0.024 wt.%) or higher, Ce_2_O_2_S became the stable phase. Therefore, to modify the Ca–Al–O inclusions in C110 steel, the appropriate amount of Ce addition was 0.01 wt.% under the present experimental conditions.

(2) The diffusion of dissolved Ce in molten steel is a restrictive step that affects the type of modified products and the growth of inclusions. When Ce alloy is added to molten steel for 5 min, the inclusions are Ce-containing mixed phase. In addition, with the increase in Ce content in the steel, the contents of Ca and Al in the inclusions decreased gradually. When the reaction time was extended to 30 min, the CaO·Al_2_O_3_ inclusions were completely modified into stable Ce_2_O_3_ and/or Ce_2_O_2_S, and the inclusions became larger.

(3) The industrial tests showed that the qualification rate of C110 thin-walled pipe for SSC resistance significantly increased from an average of 66% to 91.7% after the addition of the rare earth element. This can be attributed to the fact that the Ca–Al–O inclusions are modified into rare earth inclusions, which are much closer to the steel matrix in terms of their physical properties and deformation behavior. In addition, the rare earth inclusions can combine with S and reduce its activity during solidification, which helps to avoid the formation of CaS + Ca–Al–O duplex inclusions. Thus, the risk of cracking is eliminated or alleviated, and the SSC-resistant performance of C110 steel is improved accordingly.

## Figures and Tables

**Table 1:** Main compositions of C110 billet (wt.%).

C	Si	Mn	Cr	Mo	Als	Ca	T.O	S
0.195	0.22	0.48	0.5	0.66	0.0256	0.0027	0.0018	0.0018

**Table 2:** Amount of Ce added to the oil casing steel and the chemical composition of the sample, wt.%.

Case	Ce Alloy Addition	Als	Ca	T.O	S	Ce		
						T2	T3	T4
L–Ce	0.17 g Ce–Fe (Ce Addition 0.010 wt.%)	0.0051	0.0015	0.0028	0.0018	0.0088	0.0062	0.0057
M–Ce	0.42 g Ce–Fe (Ce Addition 0.024 wt.%)	0.0048	0.0012	0.0024	0.0021	0.0183	0.0170	0.0154
H–Ce	0.72 g Ce–Fe (Ce Addition 0.042 wt.%)	0.0050	0.0014	0.0027	0.0017	0.0387	0.0395	0.0384

**Table 3:** Possible reactions in steel after Ce addition and their standard Gibbs free energy [26,27].

Reaction Equations in Molten Steel	∆G^θ^, J/mol	No.
[Ce] + [Al] + 3[O] = CeAlO_3_	−1,366,460 + 364 T	(1)
[Ce] + 3/2[O] = 1/2Ce_2_O_3_	−715,560 + 180 T	(2)
[Ce] + [O] + 1/2[S] = 1/2Ce_2_O_2_S	−676,795 + 166 T	(3)
[Ce] + CaO·Al_2_O_3_ = CeAlO_3_ + CaO + [Al]	−123,133 − 12.52 T	(4)
[Ce] + 1/2CaO·Al_2_O_3_ = 1/2Ce_2_O_3_ + 1/2CaO + [Al]	−1,021,604 + 262 T	(5)
[Ce] + 1/2[S] + 1/2CaO·Al_2_O_3_ = 1/2Ce_2_O_2_S + [Al] + 1/2[O] + 1/2CaO	−982,839 + 247 T	(6)

**Table 4:** Interaction coefficients of elements involved in the study [26,28,29,30,31].

	C	Si	Mn	Cr	Mo	S	Al	O	Ce	Ca
O	−0.45	−0.13	−0.02	−0.04	0.0035	−0.13	−1.17	−0.17	−0.57	−515
S	0.11	0.063	−0.03	−0.011	0.0027	−0.028	0.035	−0.27	−0.23	−110
Al	0.091	0.006	0.012	0.025		0.03	0.043	−1.98	−0.04	−0.05
Ce	−0.08		0.13			−8.39	−2.25	−5.03	−0.004	−0.002
$r_{\mathrm{Al}}^{O}=39.82;\;r_{\mathrm{Al}}^{\mathrm{Al},O}=-0.028;\;r_{O}^{\mathrm{Al}}=-0.01;\;r_{O}^{\mathrm{Al},O}=-302.406;\;r_{O}^{\mathrm{Ca}}=357;\;r_{O}^{\mathrm{Ca},O}=1788$										

**Table 5:** Calculation results of atomic structure mismatch between Ce-modified products and the solidification phase.

Phase	Crystal System	*a*/nm	*c*/nm	Case	*δ*/%
δ-Fe	bcc	0.294			
Ce_2_O_3_	hex	0.389 [43]	0.606	(0001)_Ce_2_O_3__//(111) _δ-Fe_	4.8
CeAlO_3_	fcc	0.377 [44]		(100)_CeAlO_3__//(100) _δ-Fe_	7.7
Ce_2_O_2_S	hex	0.497 [43]	0.682	(0001)_Ce_2_O_2_S_//(111) _δ-Fe_	2.8
CeS	fcc	0.576 [43]		(100)_CeS_//(100) _δ-Fe_	0.3

## Data Availability

The data presented in this study are available on request from the corresponding authors.
